# Assessment of health-related quality of life in Turkish patients with facial prostheses

**DOI:** 10.1186/1477-7525-11-11

**Published:** 2013-01-26

**Authors:** Arzu Atay, Kadriye Peker, Yumushan Günay, Servet Ebrinç, Banu Karayazgan, Ömer Uysal

**Affiliations:** 1 Department of Prosthodontics, GATA Haydarpaşa Training Hospital Dental Service, Usküdar, Istanbul, Turkey; 2 Department of Dental Public Health, Faculty of Dentistry, Istanbul University, 34093 Fatih/Çapa, Istanbul, Turkey; 3 Department of Psychiyatry, GATA Haydarpaşa Training Hospital, Usküdar, Istanbul, Turkey; 4 Department of Prosthodonticsy, Dental Faculty of Baskent University, Altunizade, Istanbul, Turkey; 5 Department of Medical Statistics and Informatics, Medical School, Bezmialem Vakif University, Fatih, Istanbul, Turkey

**Keywords:** Quality of life, Facial prosthesis, Acquired facial defect, WHOQOL-BREF

## Abstract

**Background:**

Facial prostheses are intended to provide a non-operative rehabilitation for patients with acquired facial defects. By improving aesthetics and quality of life (QOL), this treatment involves reintegration of the patient into family and social life. The aim of this study was to evaluate the perception of QOL in adult patients with facial prostheses and to compare this perception with that of a control group.

**Methods:**

The study participants consisted of 72 patients, who were divided into three equal-sized groups according to the type of prosthesis (OP- orbital prosthesis, AP- auricular prosthesis, NP - nasal prosthesis) and 24 healthy control participants without any congenital or acquired deformity of face or body. Clinical and socio-demographic data were gathered from each person’s medical chart. Participants completed the Turkish version of the World Health Organization Quality of Life Instrument, Short Form (WHOQOL-BREF). Descriptive statistics, independent sample t-tests, Pearson's chi-square test, ANOVA, ANCOVA, and Pearson correlation were used to analyse the data.

**Results:**

Compared with the control participants, patients with NP scored lower on the all domains of QOL and all three patient groups had lower scores on overall QOL and its domains of physical and environmental health. Patients with OP reported significantly lower physical health scores than those with AP, while patients with NP reported significantly lower overall QOL and psychological health scores than those with AP. Female patients had lower environmental domain scores than did male patients. The patient’s age and income correlated with social relationships QOL, while the patient’s income and the age of facial prosthesis were correlated with environmental QOL.

**Conclusion:**

Patients with facial prostheses had lower scores in overall QOL, physical and environmental health domains than the control participants. Socio-demographic and clinical characteristics such as age, gender, income, localization of the defect, and age of facial prosthesis were associated with patients’ QOL. These findings may provide valuable information about the specific health needs of these patients that may affect their well-being. Further studies are needed to confirm these results. Use of the WHOQOL-BREF may provide valuable information for determining patients’ needs and priorities as well as for planning and developing comprehensive prosthetic rehabilitation programs.

## Background

In recent years, there has been growing interest in using patient reported outcomes to facilitate patient-centred care, to screen for physical and psychological problems and to monitor a patient's progress over time [1,2]. Facial disfigurement as a result of a congenital anomaly, trauma or tumour surgery can have devastating effects on the aesthetic, functional, economic, and psychosocial aspects of a person’s life [3-8].

Patients with facial defects may express unhappiness with their body image, often leading to low self-esteem, post-traumatic stress disorder symptoms and social isolation caused by stigma [4-10]. Stigma is accepted as an important social determinant of health because it may contribute to suffering, delay in appropriate help-seeking and treatment dropout and effectiveness. Thus, stigma has become a matter of particular interest for public health [3,8,11]. In this context, maxillofacial rehabilitation through prosthetic restoration is a cornerstone of efforts to restore the function and form of patients with missing or disfigured orofacial structures [12,13]. Facial prostheses, an alternative to surgery, offer non-operative rehabilitation seeking to provide satisfactory aesthetics and quality of life (QOL) and thus to facilitate reinstatement of patients in their family situation and social environment [4,5,8,9,12,14-19]. Previous studies have shown that patients with acquired facial disfigurement have greater psychosocial problems, difficulty adjusting to their facial disfigurement and more physical impairment of QOL than patients with congenital facial disfigurement [20-23]. Because of this, in a clinical setting, the identification of patients’ needs for prosthetic rehabilitation, a process which can restore QOL, is most important. Recent studies have emphasized the importance of developing additional programs to improve the quality of care and to enhance the well-being and satisfaction of patients [14-16]. The evaluation of patients’ QOL related to prosthetic rehabilitation may provide valuable information to assist the maxillofacial prosthodontic team in treatment planning, monitoring, and outcome assessment [8,9,12,14-16]. Although there are many studies examining the effects of prosthetic rehabilitation on health-related QOL in patients with maxillary defects [24-28], there are few studies investigating the health-related QOL of adult patients with facial prostheses. Recent studies have shown that implant-retained facial prostheses provide significant enhancement in patients QOL [15,18,19] and they were tolerated more easily than adhesively retained prostheses [15,19]. Patients with acquired eye and nose defects had lower health-related QOL than healthy individuals, as well as patients with acquired auricular defects [16]. To our knowledge, there is no published study evaluating the health related QOL in Turkish adult patients with facial prostheses to corroborate these findings. Health-related QOL is a multi-dimensional global construct, defined as an individual's perception of his or her position in life, in the context of the culture and value systems in which the person lives and in relation to that individual’s goals, expectations, standards and concerns [29]. Health-related QOL measures the effects of an illness and its therapy upon a person’s physical, psychological and social wellbeing, as perceived by the person himself. In Turkey, critical decisions about appropriate treatments are often made on the basis of clinicians’ intuitive judgments, because of lack of evidence about people’s perceptions of the impact of facial disfigurement and its prosthetic treatment on their health and QOL.

The primary goal of maxillofacial prosthodontists is to provide patients with the highest standards of prosthodontic care. The importance of psychosocial care for these patients is often overlooked and undervalued in current care, due to the lack of a team approach in Turkey. The evaluation of the QOL could provide valuable information for planning and monitoring of prosthetic rehabilitation, based on Turkish patients’ needs and priorities [14-16,30]. The aims of this study were to evaluate the perception of QOL in patients with different types of facial prostheses using the World Health Organization Quality of Life Instrument, Short Form (WHOQOL-BREF), and to compare the QOL of these patients with the QOL of a control group.

We hypothesized the following: (i) that patients with facial prostheses would have lower QOL scores than the control group; (ii) that significant differences would exist between patients with different types of facial prostheses in QOL scores; (iii) that patients with nasal prostheses would have greater impairment in all domains of QOL; and (iiii) that patients' socio-demographic and clinical characteristics would be associated with QOL.

## Methods

### Participants and study design

This cross-sectional study was carried out at the Department of Prosthodontics which is an out-patient clinic of GATA Haydarpasa Training Hospital, Istanbul. This hospital provides tertiary care for both military and civilian patients. The study sample consisted of 96 participants, 24 in each of three patient groups defined by type of prosthesis (OP- orbital prosthesis, AP- auricular prosthesis, NP - nasal prosthesis) and 24 control participants. The sample size estimation was calculated by online ‘Java applets for power and sample size’ software [31]. Based on a previous study conducted by Klein et al. [16], the sample size was calculated to detect a 20-point between-group difference in the WHOQOLBREF scores with 80% power at 5% type I error, assuming a standard deviation of 20 points. This required 20 subjects in each group. Assuming a 20% missing data rate, a total sample of 96 subjects was required.

Patients with facial prostheses were chosen consecutively during routine check-up visits in our hospital, between January 16 and March 23, 2012. After taking a detailed history, all patients were examined clinically by an expert clinician in the prosthodontic clinic. Patients were selected by the same clinician according to the following inclusion and exclusion criteria. The inclusion criteria were: (a) aged 18 years and over, (b) at least 6 months experience of wearing a facial prosthesis, (c) sufficient cognitive ability and an adequate level of literacy to complete the questionnaire instruments, (d) no persistent or recurrent disease, (e) a clinically and functionally acceptable prosthesis. Exclusion criteria were; (a) an extensive facial defect requiring combined prosthesis, and (b) any injuries to other parts of the body in patients with traumatic facial defects. Control participants were randomly selected from a list of 53 hospital personnel working in our hospital using a web-based random number generator (http://www.randomizer.org/). Inclusion criteria for the controls were: (a) willingness to participate in the study and to give written informed consent; (b) age 18 years or older; (c) no history of mental disorders and systemic diseases; and (d) absence of prosthesis or deformity in both face and body. Participants who did not meet the inclusion criteria were replaced with another randomly selected participant from the list until we achieved the target sample size of 24 persons for the control group.

### Procedure

The study was approved by the ethics committee at the GATA Haydarpaşa Training Hospital and was conducted in accordance with the principles of the Helsinki declaration. All subjects were informed about the nature of the study by a clinic assistant. Informed consent was obtained from each subject who agreed to participate in the study. Included in the study were 72 consecutive patients who met inclusion criteria. These patients were allocated to three groups, according to the type of the facial prosthesis, as follows: (1) group AP included patients wearing auricular prostheses; (2) group NP included patients with nasal prostheses; and (3) group OP were patients wearing orbital prostheses. Data were collected via face-to face interviews with a trained research assistant in the clinic waiting room.

### Measures

Data were collected with a questionnaire instrument with two sections. The first section comprised socio-demographic attributes (gender, age, marital status, educational level, family monthly income) and clinical data (retention type of facial prostheses, age of facial prostheses, cause of the facial defects). The second section consisted of the Turkish version of WHOQOL-BREF. To measure the QOL perception of subjects, we used the WHOQOL-BREF that was developed in a wide range of languages in different cultural settings and that yields comparable scores across cultures [32,33]. The Turkish version of the WHOQOL-BREF was validated by Eser et al. [34]. The 27- item WHOQOL-BREF Turkish version consists of two overall items measuring general QOL and health condition and 24 items that are universally adopted for the WHOQOL-BREF in four domains: physical health, psychological health, social relationships, and environment, plus one additional item that was related to Turkish background. Each item was rated on a 5- point Likert scale and the domain scores were transformed on a scale from 0 to 100 to enable comparisons to be made between domains composed of unequal numbers of items. Domains are not scored where 20% of items or more are missing, and are unacceptable where two or more items are missed except for the Environment domain, which allows two missing items [32,33]. The two general items were removed from the analyses in this study, because they did not belong to any of the four subscales.

### Data analysis

Descriptive data on frequencies, proportions, and on means and standard deviations were obtained with respect to the socio-demographic and clinical characteristics of the groups. In order to compare the groups, continuous data were analysed by means of analysis of variance (ANOVA) and categorical variables were analysed using Pearson's chi-square test. As the groups showed significant differences in age (see Table 1), the effect of age was controlled statistically. Analysis of covariance (ANCOVA) was performed for each of the WHOQOLBREF domains and for the overall QOL score, using study groups as a fixed factor and age as a covariate. Variables that had a significant F value (p≤0.05) in the ANCOVA were analysed further, with least significant difference (Bonferroni). To explore the relationship between QOL and the patients’ socio-demographic and clinical characteristics, the independent sample t-test was used for dichotomous variables and Pearson's correlation coefficient for continous variables. A p≤0.05 was considered as indicating statistical significance. The internal consistency of the WHOQOL-BREF for questions and domains was evaluated using the Cronbach reliability coefficient [35]. Statistical analyses were performed using IBM SPSS Statistics 19 for Windows (SPSS Inc., Chicago, IL, USA).

**Table 1:** Socio- demographic and clinical characteristics of study participants and comparisons between the groups

	**Patients with OP**	**Patients with AP**	**Patients with NP**	**Control group**	**P value**
	**n=24**	**n=24**	**n=24**	**n=24**	
Characteristics					
Age(years), Mean (SD)	55.83 ± 16.51	47.00 ± 14.28	61.04 ± 11.00	49.04 ± 9.49	0.001^b^
Monthly Family Income, TRY^a^, Mean (SD)	1125.00 ± 442.41	1005.00 ± 527.33	1258.33 ± 500.00	938.75 ± 445.97	0.111 ^b^
Gender (n, %)					
Female	13 (54.2)	10 (41.7)	13 (54.2)	11 (45.8)	0.771^c^
Male	11 (45.8)	14 (58.3)	11 (45.8)	13 (54.2)	
Educational level (n, %)					
≤ 8 years of schooling	9 (37.5)	11 (45.8)	9 (37.5)	5 (20.8)	0.326 ^c^
>8 years of schooling	15 (62.5)	13 (54.2)	15 (62.5)	19 (79.2)	
Marital status (n, %)					
Married	9 (37.5)	10 (41.7)	11 (45.8)	16 (66.7)	0.184 ^c^
Single, divorced, or widowed	15 (62.5)	14 (58.3)	13 (54.2)	13 (33.3)	
Retention type of facial prosthesis (n, %)					
Adhesive-retained	14 (58.3)	13 (54.2)	15 (62.5)		0.842 ^c^
Implant-retained	10 (41.7)	11 (45.8)	9 (37.5)		
Age of facial prosthesis (months), Mean (SD)	39.79±15.15	33.08±15.23	34.41±19.37		0.344 ^b^
Reason for facial defect (n, %)					
Trauma	7 (29.2)	9 (37.5)	8 (33.3)		0.829 ^c^
Tumor resection	17 (70.8)	15 (62.5)	16 (66.7)		

SD, standard deviation; OP: orbital prosthesis; AP: auricular prosthesis; NP: nasal prosthesis.

^a ^Monthly Family Income measured in Turkish lira (TRY; 1 Turkish lira ≈ 0.42 EUR).

^b ^Statistical evaluation by the one-way ANOVA.

^c ^Statistical evaluation by the Pearson’s Chi- square test.

## Results

The socio-demographic and clinical characteristics of the patients and control group are shown in Table 1. There was no significant difference between the groups in terms of sociodemographic variables, except for age (p<0.01). There was no statistically significant difference between the patient groups in terms of retention type of prosthesis, age of facial prosthesis, and the causes of the acquired facial defects (p>0.05). All patients had acquired facial defects. Facial defects were primarily the result of tumour resection (n = 48, 66.7%), and 33.3% were the result of trauma (n = 24). Adhesive-retained prostheses were used by 42 patients (58.3%). The mean age of facial prostheses was 35.76 ± 16.72 months. There were no missing data for any items on the WHOQOL-BREF. The Cronbach alpha reliability coefficient for all questions on the WHOQOL-BREF was 0.93. The coefficients for each of its domains were: physical (0.85), psychological (0.82), social relationships (0.69), and environmental (0.82). These confirm the good internal consistency of the instrument. After controlling the effect of age by ANCOVA, we found significant differences between the groups in the physical (F= 12.18, p<0.001), the psychological (F= 5.73, p<0.001), social relationships (*F* = 4.56, p = 0.002), and the environmental (F=9.58, p<0.001) domains, as well as in overall QOL (F= 12.98, p<0.001). As shown in Table 2, subsequent post hoc comparisons using the Bonferroni test revealed that all three patient groups had significantly lower scores than the control group in the physical domain. In this domain, group OP patients showed significantly lower scores than participants in group AP (p<0.05). In the psychological domain, group NP patients showed lower scores than the control group and the group AP (p<0.05). Group NP patients showed significantly lower scores than the control group in the social relationships domain (p<0.05). All three patient groups showed significantly lower scores than the control group in the environmental domain (p<0.05). All three patient groups had significantly lower scores than the control participants in overall QOL (p<0.05). Group NP patients also scored significantly lower than patients with AP on the overall QOL (p<0.05).

**Table 2:** Comparison of WHOQOL-BREF domain scores and overall QOL score, adjusted for age on 0–100 scale

**Domains**	**Patients with OP**	**Patients with AP**	**Patients with NP**	**Control group**	**F test**	**P value**	**Multiple comparisons**^**a**^
	**n=24**	**n=24**	**n=24**	**n=24**			
	**Mean ± SD**	**Mean ± SD**	**Mean ± SD**	**Mean ± SD**			
Physical	53.57±18.69	69.64±17.43	55.06±19.56	84.07±13.03	12.18	<0.001	OP vs C; AP vs C
							NP vs C; OP vs AP
Psychological	63.88±17.71	68.75±14.99	51.56±11.58	72.57±19.35	5.73	<0.001	AP vs NP; NP vs C
Social relationships	63.88±17.83	70.48±17.88	60.42±21.17	78.82±19.65	4.56	0.002	NP vs C
Environmental health	57.94±17.93	65.75±20.42	58.46±13.74	83.59±12.84	9.58	<0.001	OP vs C; AP vs C
							NP vs C
Overall QOL	58.89±11.47	68.22±16.43	56.25±13.59	80.38±8.43	12.98	<0.001	OP vs C; AP vs C
							NP vs C; AP vs NP

A high score indicates better QOL.

^a ^Comparisons (Bonferroni) for Control group (C), Patients with orbital prostheses (OP); Patients with auricular prostheses (AP); and Patients with nasal prostheses (NP); groups significant at P < 0.05.

As shown in Table 3, patients’ monthly income was positively correlated with the environmental domain (r =0.39, p =0.002) and overall QOL scores (r =0.38, p =0.003). The social relationship domain score was positively correlated with patients’ monthly income (r =0.29, p =0.023), and negatively correlated with patients’ age (r =−0.27, p =0.038). In addition, the environmental domain score was positively correlated with the age of facial prosthesis (r =0.27, p =0.04). Female patients showed significantly lower QOL than did male patients in the environmental domain of the QOL (p =0.02). Educational level, marital status, retention type of facial prosthesis, and reason for facial defect were not associated with patients’ QOL.

**Table 3:** Socio-demographic and clinical characteristics associated with QOL in patients with facial prostheses

**Characteristics**	**Overall QOL**	**Physical**	**Psychological**	**Social relationships**	**Environmental health**
**Gender (n)**^**a**^	**Mean ( SD)**	**Mean ( SD)**	**Mean ( SD)**	**Mean ( SD)**	**Mean ( SD)**
Female (30)	58.09 (13.87)	58.09 (17.11)	58.74 (16.05)	61.38 (21.49)	55.73 (19.08)
Male (30)	64.34 (16.05)	61.07 (22.84)	62.49 (18.07)	69.44 (16.71)	66.66 (16.05)
p value	0.112	0.570	0.399	0.111	0.020
Educational level (n) ^a^					
≤ 8 years of schooling (22)	60.13 (17.52)	60.71 (22.58)	59.84 (20.31)	65.53 (23.89)	57.81 (20.31)
>8 years of schooling (38)	61.84 (13.90)	58.92 (18.74)	61.07 (15.14)	65.35 (16.83)	63.15 (17.13)
p value	0.678	0.743	0.807	0.975	0.281
Marital status (n) ^a^					
Married (25)	63.33 (14.22)	63.71 (19.14)	61.99 (15.74)	63.33 (19.09)	64.00 (16.24)
Single, divorced, or widowed (35)	59.70 (15.90)	56.63 (20.46)	59.64 (18.09)	66.90 (19.95)	59.19 (19.74)
p value	0.366	0.180	0.602	0.489	0.322
Retention type of facial prosthesis (n) ^a^					
Adhesive-retained (35)	60.65 (14.99)	58.97 (19.96)	59.52 (18.45)	65.47 (19.50)	60.62 (16.72)
Implant-retained (25)	62.00 (15.77)	60.42 (20.59)	62.16 (15.11)	65.33 (19.93)	62.00 (20.80)
p value	0.738	0.785	0.558	0.978	0.778
Reason for facial defect (n) ^a^					
Trauma (20)	60.31 (15.11)	56.96 (20.83)	57.08 (18.58)	68.33 (18.65)	62.65 (15.49)
Tumor resection (40)	61.66 (15.42)	60.89 (19.81)	62.39 (16.19)	63.95 (20.00)	60.47 (19.80)
p value	0.748	0.479	0.259	0.418	0.668
Age (r)^b^	−0.14	−0.16	−0.17	−0.27	0.032
p value	0.274	0.235	0.187	0.038	0.806
Monthly family income (r) ^b^	0.38	0.24	0.25	0.29	0.39
p value	0.003	0.056	0.054	0.023	0.002
Age of facial prosthesis (r) ^b^	0.17	0.11	0.03	0.10	0.27
p value	0.200	0.399	0.836	0.44	0.04

^a ^Statistical evaluation by the independent sample t test. ^b^ Statistical evaluation by the Pearson correlation coefficient.

## Discussion

Patients’ perceptions of treatment with facial prostheses are key elements in evaluating quality of care, because measuring patient outcomes such as health-related QOL in clinical practice may provide important information for planning and evaluation of extensive maxillofacial prosthetic rehabilitation [14-16]. To our knowledge, this is the first study to examine the QOL in Turkish patients wearing facial prostheses and the first to compare the QOL of these patients with the QOL of a control group. To measure health related QOL, the WHOQOL-BREF was chosen instead of existing condition-specific measures for patients requiring extraoral craniofacial prostheses [36,37], because it is the most widely used generic instrument for such patients and allows a comparison of the investigated patient population with other patient groups or with the general population [38]. A comparative study of the QOL among patients with different types of facial prostheses and a comparison with that of healthy controls might be helpful for deciding whether QOL actually differs by type of prosthesis and to identify priorities for patient care. Confirming the first hypothesis, we found that the patients with facial prostheses had significantly lower mean scores than the controls for overall QOL, and for the physical and environmental domains. These findings are not surprising, because both the localisation of facial defects and difficulties with facial prostheses affect a person’s physical functions, such as the ability to work, basic activities of daily living, mobility, and vitality [8,15,17]. The Turkish government, civil society and disabled people’s organizations in recent years have made some efforts to create an enabling environment (for example, building accessibility, effective public transportation system, accessible information and communication) for people with disabilities. Despite these positive actions, many disabled people still face many barriers in accessing commercial and public buildings, transport, employment, health care, and education [39,40]. Only the patients in Group NP had worse scores on all domains of QOL than the controls. This may be explained by the fact that any change of the appearance of the nose can lead to social isolation, psychological distress, low self-esteem and negative body image because the nose is a prominent structure of the face and plays a very significant role in determining one's facial appearance [41]. It is known that loss of part of the face and its prosthetic restoration require social and psychological adjustment because a visible disfigurement leads to lowered self-esteem, negative self-image and social isolation for life [8,21,42,43]. There are inconsistent results in the scientific literature. Klein et al. [16] showed that the patient’s own body image is significantly altered without a restriction in the acceptance of their body by others. Newton et al. [8] reported that these persons experienced many psychological and social problems, such as negative feelings or avoiding showing their partner their face without the prosthesis. The adjustment process to disfiguring conditions and facial prosthesis are influenced by the interaction between various underlying cognitive self-schemas, and the social and cultural context [8,21,42,43]. Thus, qualitative and quantitative studies are needed to assess the impacts of these underlying factors, related to adjustment, on the patients’ QOL. We found significant differences between patient groups, confirming the second hypothesis. Group OP patients showed significantly lower scores in the physical domain than group AP. This may be explained by the fact that people with impaired vision had serious restrictions in physical activities (reading, outdoor mobility, participation in leisure activities and shopping) that were negatively related to the experience of health and vitality [17]. In addition, monocular vision and the associated compromise in depth perception may reduce the patient’s ability to clean their prosthesis with hygiene products and the quality of their hygiene [44,45]. In contrast to our findings, Klein et al. [16] found no significant differences among German patients in the physical domain. The inconsistent findings between our study and the Klein et al. study [16] may be explained by differences in existing environmental opportunities for disabled people between countries. Barrier-free buildings and mobility for disabled people are focus areas of the German Disability Discrimination Act. In addition, policies on employment and social protection in Germany have been successfully adopted to promote employment and work capacity of disabled people [46]. Legislation and standards concerning accessibility are not implemented completely in Turkey because disability and accessibility in the built environment are new issues for the country [39,40]. In overall QOL and its psychological and social domains, group NP patients had the lowest scores, followed by group OP patients, confirming the third hypothesis. This trend can be explained by fact that the nose is the most prominent part of the face since it is positioned at the centre. Patients with prosthetic noses reported more problems with the prosthesis, such as going out in hot and cold weather, playing sports and allergies [42] that affected their psychological and social well-being. Other possible explanations for this can be drawn from our experience that these patients experience difficulties in camouflaging the scar and prosthesis margins more than do other patients. To date, there is only one published study that showed no effect of the application of camouflage on the QOL in patients with facial prostheses [16]. In this study, the effects of using camouflaging on the patients’ QOL were not investigated. It will be of interest in future studies to examine the effects of the application of camouflage for prostheses on patient’s QOL.

Confirming the fourth hypothesis, a significant gender difference on the environmental health domain of QOL was found in our study. Male patients reported higher scores on the environmental domain, which includes facets pertaining to leisure, environment, transport, finance, information, home, care and safety. This may be explained by the fact that males with disabilities are still seen as the primary breadwinners in Turkish society and they have greater access to financial resources, education and employment [40,47]. Age was negatively correlated with social relationship domain of QOL and monthly family income was positively correlated with social relationship and environmental health domain and overall QOL in patients with facial prostheses. There are two possible explanations for age-related differences. Firstly, providing for the needs of older adults is a duty of children or other relatives in traditional Turkish culture. Due to the recent shift from collectivism to individualism in Turkey, the traditional extended family structure has changed and the number of older adults living independently in their own homes has begun to increase [48]. Secondly, aging can lead to decreased contact with others and change in social roles due to impaired mobility. In Turkey, individuals with higher incomes have greater opportunities for healthy living through greater access to health protecting resources such as the ability to live in safe and healthy homes, to get the best possible education and to access health care. They may also have more social support from family and friends. Other socio-demographic characteristics such as marital status and education showed no significant impact on any domain of QOL in these patients.

Among the clinical characteristics that we assessed, age of facial prosthesis was significantly associated with the environmental domain of QOL. A possible explanation for this finding is that the reactions of other people and the maintenance of the prosthesis are major concern for new users that can impair the patient’s environmental functioning [8]. Other clinical characteristics such as the reason for facial defect and retention type of facial prosthesis showed no significant impact on any domain of QOL in these patients. In contrast to our findings, Klein et al. [16] found that facial cancer patients had higher environmental domain scores than facial trauma patients. Consistent with our findings, they found no significant differences between patients with implant and adhesive retained prostheses in QOL. Future studies are needed to explore further the relationship among sociodemographic and clinical characteristics and the QOL of these patients.

There are several limitations to this cross-sectional study that should be considered when evaluating these findings. The study was conducted in only one large training hospital, limiting the generalisability of the results and the conclusions. In this study, consecutive patients with facial prostheses were compared with a control group. Significant age differences existed among the study groups and ANCOVA was used to control for the impact of this difference on QOL domain scores. Recent studies have shown that these patients’ QOL and satisfaction were associated with many clinical factors such as the localization of the facial defect, the causes of facial defects, the age of facial prostheses, and the retention mechanism of the prosthesis [15,16]. It should be noted that only consecutive patients with acquired facial defects due to trauma and tumour were included in this study. The cross-sectional design did not allow causation or changes over time in patients’ QOL to be studied. In this study, the WHOQOL-BREF was selected, based on the aims of the study. Future clinical and longitudinal studies using condition-specific measures for patients requiring extraoral craniofacial prostheses [36,37] may provide valuable information for the maxillo-prosthetic team in assessing self-perceived change of QOL in these patients. It should be noted that only the patients’ QOL was investigated in this study. There is a need for studies using qualitative and quantitative methods, as this would foster greater understanding of the psychological and emotional processes involved in adjusting to disfiguring conditions, their prosthetic restoration and the impact of these processes on patients’ QOL.

## Conclusions

Based on this preliminary study, the following conclusions can be drawn:

1. Patients with AP, OP, and NP had lower scores in the overall QOL, physical and environmental health domains than the control group, while only patients in group NP showed more psychological and social impairment than the control group. These findings highlight the need for actions to improve the QOL of these patients.

2. Clinical characteristics such as the localisation of defect and age of facial prosthesis are significantly associated with patients’ QOL. Patients with NP had the lowest scores in overall QOL and its psychological and social domains, patients with OP had the lowest score in physical and environmental health domains. New users of prostheses reported lower environmental health QOL. Although these preliminary findings may provide valuable information about the specific health needs of these patients when planning prosthetic rehabilitation programs, future studies are needed to clarify and confirm these findings.

3. Socio- demographic characteristics such as age, income, and gender should be taken into account when interpreting the patients’ QOL.

4. QOL assessment using WHOQOL-BREF may provide valuable information for determining a patient’s needs and priorities as well as for planning and developing comprehensive prosthetic rehabilitation programs.

5. The findings described in this study will be useful in the development of multidisciplinary studies, adopting both qualitative and quantitative methods to evaluate the adjustment process and its impacts on these patients’ QOL.

## Abbreviations

QOL: Quality of life; WHOQOL-BREF: The World Health Organization Quality of Life Instrument, Short Form; OP: Orbital prosthesis; AP: Auricular prosthesis; NP: Nasal prosthesis; ANCOVA: Analysis of covariance; ANOVA: Analysis of variance.

## Competing interests

The authors declare having no competing financial interest/funding for this paper.

## Authors’ contributions

AA and YG were principal investigators of this study. They contributed to the interpretation of the data and review of the manuscript. AA and KP conceptualized the study, contributed to the interpretation of the data and wrote the manuscript. SE participated in study design. BK contributed to the data collection. ÖU contributed to the study design and data analysis and calculated the required sample size. All authors read and approved the final manuscript.
